# Disparities in multidimensional psychosocial stressors by sexual minority identity among cancer survivors from the All of Us (AoU) Research Program

**DOI:** 10.21203/rs.3.rs-6884066/v1

**Published:** 2025-06-18

**Authors:** Angel Arizpe, Nikta Saeedi, Carol Y. Ochoa-Dominguez, Theresa A. Hastert, Alberto Carvajal, Sue E. Kim, Albert J. Farias

**Affiliations:** University of Southern California; University of Southern California; University of California, San Diego; Wayne State University; University of Southern California; University of Southern California; University of Southern California

**Keywords:** discrimination in medical settings, All of Us, perceived stress, cancer survivor, sexual minorities, stressors, political party

## Abstract

**Background:**

Sexual minority (SM) individuals may face discrimination and psychosocial stressors that can adversely impact their cancer care and outcomes. Therefore, we tested for disparities in psychosocial stressors by SM status among cancer survivors and explored whether observed disparities differ by governor’s political affiliation.

**Methods:**

Perceived stressors and SM status data from 2018–2022 were obtained from adult cancer survivors identified in the All of Us (AoU) data repository. We evaluated associations between self-reported SM status (heterosexual vs gay, lesbian, bisexual, or other SM minorities) and binary indicators of discrimination in medical settings (any vs. none), perceived stress (high/medium vs low), and neighborhood social cohesion (high/medium vs low) using multivariable logistic regression and stratified models adjusting for sociodemographic and clinical covariates.

**Results:**

In our cohort (N=14,806), 6.3% of survivors reported being a SM. In adjusted models, odds of reporting high/medium levels of perceived stress were 46% (95% CI: 25%, 70%) higher, and odds of low neighborhood social cohesion were 47% (95% CI: 27%, 71%) higher among SM compared to non-SM survivors. In stratified analyses (p_interaction_ 0.01), among survivors living in states with Republican governors, SM had twice the odds of experiencing discrimination in medical settings (OR: 2.31, 95% CI: 1.50, 3.71) compared to heterosexual survivors. We did not find a significant association in discrimination in the medical setting among SM living in states with Democratic governors.

**Conclusion:**

SM cancer survivors face significant disparities in reported psychosocial stressors, which may impact survivorship outcomes. Associations may differ based on broader political context.

## Précis

Sexual minority cancer survivors report significantly higher levels of perceived stress and lower neighborhood social cohesion compared to heterosexual survivors. Disparities in medical discrimination were most pronounced among those living in states with Republican governors, highlighting the influence of political context on survivorship experiences.

## Background

Approximately 4.5% of adults in the United States identify as sexual minorities (SM), those who identify as lesbian, gay, or bisexual.^1^ SM individuals experience significant health disparities compared to their heterosexual counterparts, largely due to systemic barriers in healthcare access, stigma, and discrimination.^2^ The minority stress model is a framework that suggests that chronic exposure to prejudice, social rejection, and internalized stigma contributes to worse health outcomes for SM populations.^2^ Studies have shown that SM patients often delay or avoid medical care because of prior negative experiences or fears of mistreatment from providers.^3^ In a national survey from 2011, nearly 8% of SM adults reported being denied care altogether, while many others described hostility or dismissal of their health concerns.^4^

Chronic stress from cumulative systemic discrimination has been linked to both physiological and psychological harm. For instance, discrimination-related stress has been found to be associated with elevated inflammation and cardiovascular risk.^5^ In addition, prolonged stress may lead to risky health behaviors, such as increased substance use, poor diet, and avoidance of medical care, all of which can compound negative health outcomes.^6^ These stress-related mechanisms are particularly important to consider in the context of serious or chronic illness, like cancer, where stress and behavior may directly influence disease progression and treatment outcomes.

As such, it makes sense that sexual minority cancer survivors face unique challenges that are shaped not only by the general burdens of cancer survivorship but also by the added effects of stigma, discrimination, and minority stress. There are over 18.1 million cancer survivors in the United States, many of whom face the physical, emotional, and financial burdens of ongoing treatment and monitoring.^7^ The frequency of medical appointments and the long-term treatments required for survivorship may exacerbate chronic stressors like societal marginalization, particularly when SM survivors are reluctant to engage with healthcare due to prior experiences of discrimination.^8^ Older SM survivors—particularly gay and bisexual men—are more likely to live alone and report social isolation^9,^ which has been associated with later-stage diagnoses and poorer cancer survival outcomes^10^.

Beyond individual experiences, the broader political and social climates in which SM survivors reside may influence their access to care and psychosocial well-being, as state-level policies and societal attitudes can create environments that either mitigate or exacerbate challenges for SM populations. While it is well established that stigma, discrimination, and psychosocial stressors negatively impact healthcare access and outcomes for SM individuals, there is limited understanding of these associations in the context of cancer survivorship. Furthermore, to our knowledge, no studies have assessed how these disparities differ based on broader political environments. Thus, this study aims to estimate associations between sexual minority status and psychosocial stressors among cancer survivors from the All of Us program and explore whether these associations differ by state socio-political climates as measured by the state governor’s political affiliation.

## Methods

### Data Collection and Sample

We assessed cross-sectional survey data from May 2018 to July 2022 from “All of Us” (AoU). Participants enrolled in the AoU program signed a consent form for data collection following the Declaration of Helsinki protocol. Data for this study were de-identified and made available to AoU-approved researchers. The All of Us program was approved by the National Institutes of Health (NIH) Institutional Review Board (IRB).

**Figure 1:**
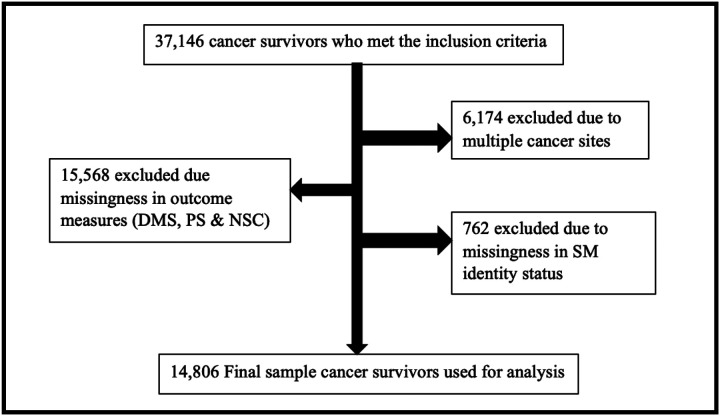
Decision tree describing the cohort’s inclusion and exclusion criteria.

Our cohort included participants who reported that they were ever told by their healthcare provider that they had/have cancer. We excluded participants with missing data on self-reported discrimination in the medical setting, social neighborhood cohesion, and perceived stress scales; those with multiple cancers, and missing sexual orientation status (Fig. 1).

### Measures

#### Demographics and covariates:

Demographic characteristics included in our study were continuous age, biological sex (male vs. female), race/ethnicity (non-Hispanic White vs. categories: non-Hispanic Asian, non-Hispanic Black, Hispanic, and, Other [includes: more than one race, another race, and none of these]), marital status (married [includes: living with a partner] vs single [includes: single, divorced, widowed, and separated]), active cancer treatment (yes vs. no), nativity status (US-born vs foreign-born) and socioeconomic barrier index (SES): five SES factors (education[≤ High school], income[≤$35K, which are those in the lowest quantile], insurance[none], housing[rent/other], and employment status[unemployed]) each dichotomized to create a composite measure as detailed in a previous study ranging from 0 to 5, that were truncated to 3 + due to sparsity. Higher scores in this SES index indicate higher SES barriers.^11^

### Exposure

#### Sexual Minorities (SM)

Using a single question that asked participant to describe the best respresention of how they think of themselves, we created a binary indicator of SM identity where those who self-identified as heterosexual were coded as heterosexual and those who self-identified as bisexual, gay, lesbian, or other (e.g., queer, asexual, two-sprit, polusexual, omnisexual, sapiosexual or pansexual) were coded as SM.

### Outcomes

*Discrimination in the Medical Settings (DMS)* is an adapted 7-items^12^ scale from the Everyday Discrimination Scale (EDS)^13^, that assesses the participants’ prior treatment experiences while getting healthcare services. Participants were asked, “How often do any of these (perceived discriminatory events) happen to you when you go to a doctor’s office or other health care provider? Example items include “You feel like a doctor or nurse is not listening to what you were saying”, “A doctor or nurse acts as if he or she thinks you are not smart.”, “A doctor or nurse acts as if he or she is afraid of you”. Responses were measured on a 5-point Likert scale, ranging from never (0) to always (4). We created a dichotomized indicator of never vs. any DMS, where if participants selected never having experienced DMS in all seven items, they were then coded as never and were set as the reference. Similar methods of dichotomizing each question have been assessed previously using this measure.^14^

*Neighborhood Social Cohesion (NSC)* was measured using the 4-item scale, which aims to quantify an individual’s perception of their neighborhood and experiences with trust and social relationships with those around the neighborhood they live in^15^, which are important to access resources and buffer stress. Example items include “People in my neighborhood generally get along with each other” and “People in my neighborhood share the same values”. Responses were measured on a 5-point Likert scale, which ranged from “strongly agree (1)” to “strongly disagree (5)”. We created a summed score from those questions that ranged from 4–20, where we then created a binary indicator that was categorized as low NSC, those at or above the median score, and having better NSC, those below the median score, similar to how other studies have measured it as a binary indicator.^16^

Perceived Stress (PS) was measured using the 10-item Perceived Stress Scale^17^, which asked participants about their feelings and thoughts in the past month. Example items include “In the last month, how often have you been upset because of something that happened unexpectedly?”, “In the last month, how often have you felt difficulties were piling up so high that you could not overcome them?”. Responses were measured on a 5-point Likert scale, from never to very often. We created a summed score that ranged from 0–40, where we created a binary indicator using recommended cutoffs, where we categorized low PS for scores 0–13 and high/moderate to scores ≥ 14.^17^

### Moderator

Using data from the National Governor’s Association (2023)^18^, we created one binary indicator of the governor’s political affiliation as Democrat or Republican. We used self-reported state residential information and assigned individuals to their respective governor’s political affiliation. For example, if they resided in a state with a Democratic governor, they were categorized as living in a Democratic governor’s state.

### Statistical Methods

Descriptive statistics used chi-square or Mann-Whitney U tests to determine the association of all the variables with the exposure (SM) and outcomes (DMS, NSC, PS) separately. Multivariable logistic regression models assessed whether SM identity was associated with our outcomes of interest. Interaction terms were included in the models to assess whether the SM and outcomes differed by the governor’s political affiliation (SM*governor political affiliation). Covariates included in multivariable models were age, sex, nativity, SES barriers index, 2023 Governor’s political party affiliation, PS, DMS, or NSC, and active treatment status. All statistical analyses were performed using R Jupyter Notebooks accessed via the “All of Us” workbench and using a significance level at alpha < 0.05. Odds ratios (ORs) with 95% confidence intervals (CI) and p-values were reported.

## Results

Our final analytical sample consisted of 14,806 cancer survivors with a median age of 69 (Interquartile range [IQR(Q1, Q3)] = 59.9, 74.6) years. Most participants identified as non-Hispanic White as their race/ethnicity (88%) and reported their biological sex as female (61%). The majority of cancer survivors reported being married (68%), US-born (93%), and having experienced any DMS (72%). Approximately half reported low PS (52%) and low NSC (49.5%) (Table 1). Cancer survivors who self-identified as SM had a higher prevalence of ever experiencing DMS (80% vs 72%), medium/high PS (62% vs 47%), and low NSC (64% vs 48%) compared with heterosexual survivors (Table 2).

### SM and Stressors

Adjusting for the state governor’s political party and other covariates, results from the multivariable models showed that compared to heterosexual cancer survivors, those who were SM had a 34% (aOR = 1.36, 95% CI:1.15, 1.63), 46% (aOR = 1.46, 95% CI:1.26, 1.72) and 47% (aOR = 1.47, 95% CI:1.27, 1.71) greater likelihoods of reporting any DMS, high/moderate PS, and low NSC respectively (Table 3).

In models stratified by the political party affiliation of the state’s governor, we observed no difference in associations between SM identity and perceived stress (p_interaction_ = 0.96) or neighborhood social cohesion (p_interaction_ = 0.97). Associations between SM identity and experiences of medical discrimination were much stronger for cancer survivors living in states with Republican governors (aOR: 2.31, 95% CI: 1.50, 3.71) compared with survivors living in states with Democratic governors (aOR: 1.20, 95% CI: 0.99, 1.45; p_interaction_ 0.01) (Table 4).

## Discussion

Using data from the All of Us Research Program, this study found that sexual minority cancer survivors had significantly higher odds of experiencing discrimination in medical settings (DMS), moderate/high perceived stress (PS), and low social cohesion compared to their non-sexual minority counterparts. While state governor party affiliation did not significantly moderate the associations of SM identity status with perceived stress or neighborhood social cohesion, we observed a significant interaction among those residing in a state with a Republican governor and SM identity status and discrimination in the medical setting. Specifically, SM identity was associated with more than twice the odds of experiencing DMS compared to heterosexual cancer survivors living in states with Republican governors, while SM identity was associated with a non-significant 20% higher odds of DMS among survivors living in states with Democratic governors. These findings highlight the disproportionate burden of psychosocial stressors faced by SM cancer survivors and suggest that broader sociopolitical factors may play a role in shaping these experiences.

### Discrimination in the Medical Setting

Our findings indicated that sexual minority cancer survivors had higher odds of reporting discrimination in medical settings. Discrimination and stigma contribute to social isolation, and they also discourage help-seeking behaviors, which can lead to delays in medical care and worse health outcomes. Studies have shown that sexual minority individuals who perceive lower levels of community support are less likely to engage in routine healthcare and cancer screenings, thereby increasing their risk of late-stage diagnoses and poorer prognoses.^3^ Prior research has established that experiences of discrimination in medical settings can lead to medical mistrust and avoidance of healthcare, ultimately resulting in delayed diagnoses and poorer prognoses for sexual minority individuals.^19^ Additionally, stress from discrimination has been linked to chronic physiological dysregulation, resulting in higher levels of inflammation and cardiovascular risk, further exacerbating health disparities.^20^ With respect to cancer survivorship, lower levels of social cohesion and support may further exacerbate these disparities, as strong social networks have been shown to improve adherence to treatment and overall health outcomes.^21^

### Social Cohesion among Sexual Minority Cancer Survivors

Our findings suggest that sexual minority cancer survivors have significantly higher odds of reporting low neighborhood social cohesion compared to their heterosexual cancer survivor counterparts. This finding aligns with prior research suggesting that SM experience differences in their perceptions of social cohesion and reinforces the importance of social networks for this vulnerable group. One study found that lesbian, gay, and bisexual (LGB) adults were less likely to feel that their neighborhood was close-knit, to trust their neighbors, or to believe neighbors helped each other out, even after adjusting for socio-demographic characteristics, living arrangements, health status, region, and neighborhood tenure.^22^ Although we found lower levels of neighborhood social cohesion within our study cohort, neighborhood social cohesion is also associated with fewer psychological symptoms and serves as a protective factor against negative health outcomes, including myocardial infarction and stroke.^23,24^ One study notes that even perceived social support may also be more critical than actual support available or received in terms of quality of life outcomes, such as reduced depressive symptoms, lower levels of distress, and improved mental health.^25,26^

### State Governor Party Affiliation

Our findings suggest that while Republican state Governor’s party affiliation significantly moderated the association between SM identity status and discrimination in medical settings (DMS) for sexual minority cancer survivors, it did not moderate the associations between SM identity status and perceived stress or neighborhood social cohesion. This aligns with research that specific policy environments can directly influence how SM individuals experience healthcare. State policies such as employment nondiscrimination protections can protect against minority stress, while exclusionary measures like antigay marriage amendments exacerbate minority stress by limiting access to resources and increasing exposure to stigma.^27^ Similarly, the presence or absence of nondiscrimination and religious exemption laws is closely tied to mental health outcomes among SM adults, wherein states without these protections or with broad exemptions are significantly associated with higher levels of anxiety and depression.^28^ In our study, the link between gubernatorial party affiliation and discrimination in healthcare likely reflects a similar mechanism, as Republican governors can play a key role in determining whether protective or harmful policies are enacted. This would make medical discrimination more directly tied to a state’s political leadership.

The lack of association by gubernatorial party affiliation between SM minority identity and PS or NSC in our study, however, may reflect a more multifactorial nature of PS and NSC. PS and NSC are also likely shaped by personal, community, and cultural factors that extend beyond state-level leadership, as these outcomes develop over longer periods and may be more of a response to nationwide sociocultural trends or movements. While policies matter, the ways through which they influence outcomes, such as their visibility, enforcement, and cumulative exposure over time, are complex and not always experienced uniformly.^28^ Ultimately, our findings highlight that while political leadership may be a key driver of discrimination in clinical settings, psychosocial outcomes like stress and neighborhood social cohesion are broader and may require more nuanced, qualitative models to fully understand how they are associated with a state’s political environment.

### Strengths, Limitations, and Future Direction

To our knowledge, this is the largest study on sexual minority identity and psychosocial stressors among cancer survivors in the US. Harnessing the All of Us research program, we assessed the independent associations of sexual minority identity status and different types of stressors (DMS, PS, NSC) in a large sample of U.S. adult cancer survivors. A key strength of this study is the use of a large dataset that enhances the sample size of sexual minorities and thus the generalizability of our findings, as we have a range of survivors from geographically diverse areas in the US. By incorporating state-level political context, to our knowledge, this is the first study to examine state-level political context in SM cancer survivor experiences, addressing a critical knowledge gap in SM and cancer survivor research. Furthermore, the inclusion of multiple psychosocial measures allows for a more nuanced analysis of distinct aspects of social determinants of health, providing a comprehensive understanding of their impact on this population.

However, this study is not without limitations. We excluded a slightly higher proportion of racial/ethnic minorities and those with two or three or more SES barriers because they did not have complete information on psychosocial stressors, which could have introduced a potential for selection bias towards the null. Thus, as the AoU programs continue to enroll participants and those enrolled complete surveys, future studies should reassess this relationship to better understand the potential impact sexual minority cancer survivors could experience.

## Conclusion

The All of Us Research Program data allowed us to investigate the independent associations between sexual minority status and different types of stressors among a large sample of cancer survivors. We further explored whether these associations differed by the state governor’s political affiliation. We found that identifying as a sexual minority is associated with increased likelihood of experiencing any DMS, medium/high PS, and low NSC, compared to their heterosexual counterparts. Moreover, there were differences in the governor’s political affiliation in the sexual minority status and DMS relationship. We found that, residing in a state governed by a Republican governor, sexual minority cancer survivors had increased odds of experiencing any DMS compared to their heterosexual counterparts. No differences were found among those who resided in a state led by a Democratic governor. Our findings could help guide healthcare systems in certain states to promote the inclusivity and acceptance of sexual minority survivors to minimize the potential to forgo medical care due to prior discriminatory experiences.

## Figures and Tables

**Table 1:** Demographic characteristics of AoU cancer survivors by sexual orientation status

	Heterosexual	Sexual Minority	*p*	Total
	(N = 13,867)	(N = 939)		(N = 14,806)
Race/Ehtnicity			0.02	
Non-Hispanic White	12,244 (88.3%)	826 (88.0%)		13,070 (88.3%)
Hispanic	670 (4.8%)	58 (6.2%)		728 (4.9%)
Non-Hispanic Black	505 (3.6%)	30 (3.2%)		535 (3.6%)
Non-Hispanic Asian	> 100 (> 0.5%)	≤ 20 (< 1.0%)		145 (1.0%)
Other	> 80 (> 0.5%)	≤ 20 (< 1.5%)		104 (0.7%)
Missing	> 180 (> 1.0%)	≤ 20 (< 1.0%)		224 (1.5%)
Sex			***	
Male	> 5,000 (> 35.0%)	< 500 (< 55.0%)		5.675 (38.3%)
Female	8,607 (62.1%)	437 (46.5%)		9.044 (61.1%)
Missing	> 80 (> 0.5%)	≤ 20 (< 1.0%)		87 (0.6%)
Age			***	
Mean (SD)	66.2 (11.7)	60.7 (13.9)		65.8 (11.9)
Median [Q1, Q3]	68.4 [59.7, 74.2]	63.6 [52.5, 70.6]		67.9 [59.6, 74.4]
Income Status			***	
Q1 (lowest income)	2,997 (21.6%)	321 (34.2%)		3,318 (22.4%)
Q2-Q5	10,870 (78.4%)	618 (65.8%)		11,488 (77.6%)
Marital Status			***	
Married	> 9,500 (> 65.0%)	< 600 (< 55.0%)		10,079 (68.1%)
Single	4,205 (30.3%)	415 (44.2%)		4,620 (31.2%)
Missing	> 80 (> 0.5%)	≤ 20 (1.4%)		107 (0.7%)
Education Status			0.65	
Some College +	> 12,000 > (90.0%)	< 900 (< 95.0%)		13,558 (91.6%)
≤ HS	1,077 (7.8%)	67 (7.1%)		1144 (7.7%)
Missing	> 80 (> 0.5%)	≤ 20 (0.4%)		104 (0.7%)
Insurance Status			0.36	
Insured	13,648 (98.4%)	918 (97.8%)		14,566 (98.4%)
Uninsured	> 100 (> 0.5%)	≤ 20 (< 2.0%)		141 (1.0%)
Missing	> 80 (> 0.5%)	≤ 20 (<1.0%)		99 (0.7%)
Nativity			0.63	
US-Born	> 12,500 (> 90.0%)	< 900 (< 95.0%)		13,831 (93.4%)
Foreign-Born	864 (6.2%)	50 (5.3%)		914 (6.2%)
Missing	> 45 (> 0.1%)	≤ 20 (< 1.0%)		61 (0.4%)
Housing Status			***	
Own	> 11,000 (> 80.0%)	< 600 (< 65.0%)		11,821 (79.8%)
Rent/Other Arrangement	2,493 (18.0%)	346 (36.8%)		2,839 (19.2%)
Missing	> 100 (> 0.5%)	≤ 20 (< 1.0%)		146 (1.0%)
Employment Status			***	
Employed	> 12,000 (> 90.0%)	< 800 (< 85.0%)		13,434 (90.7%)
Unemployed	1,127 (8.1%)	136 (14.5%)		1,263 (8.5%)
Missing	> 80 (> 0.5%)	≤ 20 (< 1.0%)		109 (0.7%)
Governor Political Affiliation			0.15	
Democrat	> 11,000 (> 80.0%)	< 800 (< 80.0%)		11.858 (80.1%)
Republican	2,709 (19.5%)	205 (21.8%)		2.914 (19.7%)
Missing	> 20 (> 0.1%)	≤ 20 (< 0.1%)		34 (0.2%)
Active Treatment			0.02	
No	> 10,000 (> 70.0%)	< 800 (< 80.0%)		10.911 (73.7%)
Yes	3,643 (26.3%)	206 (21.9%)		3,849 (26.0%)
Missing	> 20 (> 0.1%)	≤ 20 (< 1.0%)		46 (0.3%)
Socioeconomic Barrier			***	
0	9,497 (68.5%)	487 (51.9%)		9,984 (67.4%)
1	2,795 (20.2%)	244 (26.0%)		3,039 (20.5%)
2	973 (7.0%)	111 (11.8%)		1,084 (7.3%)
3+	602 (4.3%)	97 (10.3%)		699 (4.7%)
Discrimination in Medical Settings			***	
Never	3,941 (28.4%)	190 (20.2%)		4,131 (27.9%)
Any	9,926 (71.6%)	749 (79.8%)		10,675 (72.1%)
Perceived Stress			***	
Low	7,385 (53.3%)	354 (37.7%)		7,739 (52.3%)
Medium/High	6,482 (46.7%)	585 (62.3%)		7,067 (47.7%)
Social Neighborhood Cohesion			***	
Better	7,149 (51.6%)	334 (35.6%)		7,483 (50.5%)
Low	6,718 (48.4%)	605 (64.4%)		7,323 (49.5%)

Notes:

SES = Socioeconomic, Married includes living with a partner,

Single includes Divorced, Widowed, and Separated.

Per “All of Us” data use agreement policy, groups < 20 participants are shown as ≤ 20 (%) with a corresponding > (%) category to prevent deriving counts < 20 from other values.

No all percentages equal to 100. Significant P-values

*** <0.001,

** <0.01,

* <0.05

Chi-square or Fisher tests were performed to obtain p-values (*p*)

Income: Lowest Quintile: includes individuals with income of ≤ $35K

**Table 2:** Demographic characteristics of AoU cancer survivors by types of stress indicators (N = 14,806)

	Discrimination in Medical Settings	*p*	Perceived Stress	*p*	Social Neighborhood Cohesion	*p*
	Any		Medium/High		Low	
	(N = 10,675) 72.1%		(N = 7,067) 47.7%		(N = 7,323) 49.5%	
Race/Ethnicity		0.35		***		***
Non-Hispanic White	> 9,000 (> 70.0%)		6,085 (46.6%)		6,254 (47.9%)	
Hispanic	529 (72.2%)		454 (62.4%)		456 (62.6%)	
Non-Hispanic Black	392 (73.3%)		285 (53.7%)		364 (68.0%)	
Non-Hispanic Asian	107 (73.8%)		80 (55.2%)		74 (51.0%)	
Other	< 100 (< 85.0%)		66 (63.5%)		64 (61.5%)	
Missing	160 (71.4%)		97 (43.3%)		111 (49.6%)	
Sex		***		***		0.16
Male	3,764 (66.3%)		2,167 (38.2%)		2,740 (48.3%)	
Female	6,848 (75.7%)		4,853 (53.7%)		4,541 (50.2%)	
Missing	63 (72.6%)		47 (54.0%)		42 (48.3%)	
Sexual Minority Status		***		***		***
Heterosexual	9,926 (71.6%)		6,482 (46.7%)		6,718 (48.4%)	
Sexual Minority	749 (79.8%)		585 (62.3%)		605 (64.4%)	
Age		***		***		***
Mean (SD)	65.2 (12.1)		62.5 (12.9)		64.6 (12.5)	
Median [Q1, Q3]	67.5 [58.6, 73.6]		64.6 [54.6, 72.0]		66.8 [57.6, 73.6]	
Income		***		***		***
Q1 (lowest income)	2,560 (77.2%)		1,998 (60.2%)		2,080 (62.7%)	
Q2-Q5	8,115 (70.6%)		5,069 (44.1%)		5,243 (45.6%)	
Marital Status		***		***		***
Married	7,112 (70.6%)		4,518 (44.8%)		4,531 (45.0%)	
Single	3,483 (75.4%)		2,496 (54.0%)		2,733 (59.2%)	
Missing	80 (74.8%)		53 (49.5%)		59 (55.1%)	
Education		0.04		***		***
Some College +	9,819 (72.4%)		6,361 (46.9%)		6,589 (48.6%)	
≤ HS	786 (68.7%)		652 (57.0%)		683 (59.7%)	
Missing	70 (67.3%)		54 (51.9%)		51 (49.0%)	
Insurance Status		0.35		***		***
Insured	10,490 (72.0%)		6,913 (47.5%)		7,178 (49.3%)	
Uninsured	107 (76.0%)		95 (67.4%)		86 (61.0%)	
Missing	78 (78.8%)		59 (59.6%)		59 (59.6%)	
Nativity Status		0.42		0.86		***
USA-born	9,979 (72.1%)		6,589 (47.6%)		6,777 (49.0%)	
Non-US-born	647 (70.8%)		449 (49.1%)		518 (56.7%)	
Missing	< 50 (80.3%)		29 (47.5%)		28 (45.9%)	
Housing Status		***		***		***
Own	8,393 (71.0%)		5,168 (43.7%)		5,284 (44.7%)	
Rent/Other Arrangement	2,171 (76.5%)		1,823 (64.2%)		1,957 (68.9%)	
Missing	111 (76.0%)		76 (52.1%)		82 (56.2%)	
Employment Status		***		***		***
Employed	9,576 (71.3%)		6,085 (45.3%)		6,427 (47.8%)	
Unemployed	1,025 (81.2%)		921 (72.9%)		826 (65.4%)	
Missing	74 (67.9%)		61 (56.0%)		70 (64.2%)	
Governor Political Affiliation		0.18		0.36		***
Democrat	> 8,400 (> 70.0%)		> 5,500 (> 45.0%)		> 5,500 (> 75.0%)	
Republican	2,149 (73.7%)		1,434 (49.2%)		1,507 (73.2%)	
Missing	≤ 20 (< 75.0%)		≥ 20 (< 50.0%)		≥ 20 (> 60.0%)	
Active Treatment		0.13		0.21		0.89
No	> 7,500 (> 70.0%)		5,126 (47.0%)		5,376 (49.3%)	
Yes	2,719 (70.6%)		1,920 (42.9%)		1,925 (50.0%)	
Missing	< 25 (< 70.0%)		21 (45.7%)		22 (47.8%)	
Socioeconomic Barrier		***		***		***
0	7,034 (70.5%)		4,167 (41.7%)		4,315 (43.2%)	
1	2,252 (74.1%)		1,626 (53.5%)		1,741 (57.3%)	
2	835 (77.0%)		731 (67.4%)		719 (66.3%)	
3+	554 (79.3%)		543 (77.7%)		548 (78.4%)	
Discrimination in Medical Settings				***		***
Never	-		1,213 (29.4%)		1,629 (39.4%)	
Any	-		5,854 (54.8%)		5,694 (54.4%)	
Perceived Stress		***				***
Low	4,821 (62.3%)		-		3,202 (41.4%)	
Medium/High	5,854 (82.8%)		-		4,121 (58.3%)	
Social Neighborhood Cohesion		***		***		
Better	2,901 (79.8%)		2,275 (62.6%)		-	
Low	7,774 (69.6%)		4,792 (42.9%)		-	

Notes:

SES = Socioeconomic, Married includes living with a partner,

Single includes Divorced, Widowed, and Separated.

Per “All of Us” data use agreement policy, groups < 20 participants are shown as ≤ 20 (%) with a corresponding > (%) category to prevent deriving counts < 20 from other values.

No all percentages equal to 100

Chi-square or Fisher tests were performed to obtain p-values (*p*)

Significant P-values

*** <0.001,

** <0.01,

* <0.05

Income: Lowest Quintile: includes individuals with income of ≤ $35K

**Table 3:** Multivariable Association of being a sexual minority and stressors among cancer survivors from the All of Us Research Program (N = 14,276)

	Any Discrimination in Medical Settings	Medium/High Perceived Stress	Low Neighborhood Social Cohesion
	aOR (95% CI)	aOR (95% CI)	aOR (95% CI)
Variables			
Sexual Orientation			
Heterosexual	Ref	Ref	Ref
Sexual Minority	**1.34 (1.13–1.60)**	**1.46 (1.25–1.70)**	**1.47 (1.27–1.71)**
Race/Ethnicity			
Non-Hispanic White	Ref	Ref	Ref
Hispanic	**0.82 (0.68–0.99)**	1.17 (0.97–1.40)	**1.26 (1.06–1.50)**
Non-Hispanic Black	0.89 (0.72–1.10)	0.74 (0.61–0.91)	**1.63 (1.33–1.99)**
Non-Hispanic Asian	1.04 (0.70–1.57)	1.13 (0.78–1.64)	0.92 (0.65–1.34)
Other	1.63 (0.98–2.87)	1.48 (0.96–2.32)	1.36 (0.90–2.08)
Sex			
Male	Ref	Ref	Ref
Female	**1.37 (1.27–1.49)**	**1.44 (1.33–1.55)**	0.84 (0.78–0.91)
Age	1.00 (0.99–1.00)	**0.96 (0.96–0.97)**	**0.99 (0.99–1.00)**
Governor Political Afilliation			
Democrat	Ref	Ref	Ref
Republican	**1.11 (1.01–1.22)**	0.99 (0.91–1.09)	1.04 (0.95–1.13)
Nativity Status			
US-Born	Ref	Ref	Ref
Non-US-Born	0.94 (0.80–1.12)	**0.83 (0.71–0.98)**	**1.22 (1.23–1.44)**
Active treatment			
No	Ref	Ref	Ref
Yes	**0.89 (0.82–0.97)**	**1.13 (1.04–1.23)**	0.99 (0.92–1.08)
Socioeconomic Barrier			
0	Ref	Ref	Ref
1	0.97 (0.88–1.07)	**1.36 (1.24–1.49)**	**1.51 (1.38–1.65)**
2	0.94 (0.80–1.11)	**2.13 (1.83–2.48)**	**1.87 (1.62–2.16)**
3+	0.90 (0.99–1.12)	**2.87 (2.33–3.54)**	**2.99 (2.45–3.66)**

Notes

Adjusted for governorship race, SES barriers, age, marital status, born, active treatment, biological sex, social cohesion, perceived stress

aOR = adjusted Odds Ratios, CI = confidence interval

Bolded represent statistical significance

**Table 4:** Multivariable Association of being a sexual minority and discrimination in medical settings by political party residence among cancer survivors from the All of Us Research Program (N = 14,276)

	Democratic Gov (n = 11456)	Republican Gov (n = 2920)
Heterosexual	Ref	Ref
Sexual Minority	1.20(0.99–1.45)	**2.31(1.50–3.71)**

Notes: Adjusted for race, SES barriers, age, marital status, born, active treatment, biological sex, social cohesion, perceived stress

aOR = adjusted Odds Ratios, CI = confidence interval

Bolded represent statistical significance

## Data Availability

This study used data from the All of Us data resource. The interpretation and reporting of these data are the sole responsibility of the authors. The data is publicly available with approval for use from the NIH All of Us research program on the workbench.

## References

[1] Gates GJ. (2011). How many people are lesbian, gay, bisexual, and transgender? The Williams Institute, UCLA School of Law. Retrieved from https://williamsinstitute.law.ucla.edu/wp-content/uploads/How-Many-People-LGBT-Apr-2011.pdf.

[2] Meyer IH. Prejudice, social stress, and mental health in lesbian, gay, and bisexual populations: conceptual issues and research evidence. Psychol Bull. 2003;129(5):674–97. 10.1037/0033-2909.129.5.674. 12956539 PMC2072932

[3] Tabaac AR, Solazzo AL, Gordon AR, Austin SB, Guss C, Charlton BM. Sexual orientation-related disparities in healthcare access in three cohorts of U.S. adults. Prev Med. 2020;132:105999. 10.1016/j.ypmed.2020.105999. Epub 2020 Jan 22. 31981643 PMC8312312

[4] Lambda Legal. (2010). When health care isn’t caring: Lambda Legal’s survey on discrimination against LGBT people and people living with HIV. Retrieved from https://legacy.lambdalegal.org/sites/default/files/publications/downloads/whcic-report_when-health-care-isnt-caring.pdf

[5] Saban KL. (2018). Perceived discrimination is associated with the inflammatory response to acute laboratory stress in women at risk for cardiovascular disease. Brain, Behavior, and Immunity, 73, 625–632. https://doi.org/info:doi/. 30012518 10.1016/j.bbi.2018.07.010 PMC6129426

[6] Jackson JS, Knight KM, Rafferty JA. Race and unhealthy behaviors: Chronic stress, the HPA axis, and physical and mental health disparities over the life course. Am J Public Health. 2010;100(5):933–9. 10.2105/AJPH.2008.143446. 19846689 PMC2853611

[7] American Cancer Society. (2020). Cancer Facts & Figs. 2020. Atlanta: American Cancer Society. Retrieved from https://www.cancer.org/research/cancer-facts-statistics/all-cancer-factsfigures/cancer-facts-figures-2020.html

[8] Margolies L, Brown CG. Current State of Knowledge About Cancer in Lesbians, Gay, Bisexual, and Transgender (LGBT) People. Semin Oncol Nurs. 2018;34(1):3–11. Epub 2017 Dec 25. 29284587 10.1016/j.soncn.2017.11.003

[9] Ulrike Boehmer. LGBT Populations’ Barriers to Cancer Care, Seminars in Oncology Nursing, Volume 34, Issue 1. (2018). Pages 21–29. ISSN 0749–2081. 10.1016/j.soncn.2017.11.002 29338894

[10] Karen I, Fredriksen-Goldsen H-J, Kim SE, Barkan A, Muraco. and Charles P. Hoy-Ellis: Health Disparities Among Lesbian, Gay, and Bisexual Older Adults: Results From a Population-Based Study. Am J Public Health 103, 18021809, 10.2105/AJPH.2012.301110 PMC3770805 23763391

[11] Arizpe A, Navarro S, Ochoa-Dominguez CY, Rodriguez C, Kim SE, Farias AJ. Nativity differences in socioeconomic barriers and healthcare delays among cancer survivors in the All of Us cohort. Cancer Causes Control September. 2023. 10.1007/s10552-023-01782-z. PMC10787892 37679534

[12] Peek ME, Nunez-Smith M, Drum M, Lewis TT. Adapting the Everyday Discrimination Scale to Medical Settings: Reliability and Validity Testing in a Sample of African American Patients.; 2011. PMC3350778 22428358

[13] Williams DR, Yu Y, Jackson JS, Anderson NB. Racial Differences in Physical and Mental Health Socio-Economic Status, Stress and Discrimination.; 1997. 10.1177/135910539700200305 22013026

[14] Benjamins MR, Middleton M. Perceived discrimination in medical settings and perceived quality of care: A population-based study in Chicago. PLoS ONE. 2019;14(4):e0215976. 10.1371/journal.pone.0215976. 31022267 PMC6483224

[15] Bateman LB, Fouad MN, Hawk B, Examining Neighborhood Social Cohesion in the Context of Community-based Participatory Research: Descriptive Findings from an Academic-Community Partnership. Ethn Dis. 2017;27(Suppl 1):329. 10.18865/ed.27.S1.329. 29158658 PMC5684777

[16] Yi SS, Trinh-Shevrin C, Yen IH, Kwon SC. Racial/Ethnic Differences in Associations Between Neighborhood Social Cohesion and Meeting Physical Activity Guidelines, United States, 2013–2014. Prev Chronic Dis. 2016;13:160261. 10.5888/pcd13.160261. PMC5145691 27930284

[17] Cohen S, Karmarck T, Mermelstein R. A global measure of perceived stress. J Health Soc Behav. 1983;24(4):385–96. 6668417

[18] KFF. State Political Parties. https://www.kff.org/other/state-indicator/state-political-parties/?activeTab=map&currentTimeframe=0&selectedDistributions=governor-political-affiliation&sortModel=%7B%22colId%22:%22Governor%20Political%20Affiliation%22,%22sort%22:%22asc%22%7D.

[19] Rastegar P, Cai L, Langhinrichsen-Rohling J. Racial Discrimination as a Traumatic Bedrock of Healthcare Avoidance: A Pathway Through Healthcare Institutional Betrayal and Mistrust. Healthc (Basel). 2025;13(5):486. 10.3390/healthcare13050486. PMC11899034 40077048

[20] Saban KL. (2018). Perceived discrimination is associated with the inflammatory response to acute laboratory stress in women at risk for cardiovascular disease. Brain, Behavior, and Immunity, 73, 625–632. https://doi.org/info:doi/. 30012518 10.1016/j.bbi.2018.07.010 PMC6129426

[21] Rehman R, Solorzano G, Heist R, Thompson SN, Badawi M. Predictors of poor adherence to follow-up care in survivors of childhood cancer. ONCOLOGY. 2022;36(6):350–4. 10.46883/2022.25920964. 35723943

[22] Henning-Smith C, Gonzales G. Differences by Sexual Orientation in Perceptions of Neighborhood Cohesion: Implications for Health. J Community Health. 2018;43. 10.1007/s10900-017-0455-z. 29222737

[23] Kim ES, Hawes AM, Smith J. Perceived neighbourhood social cohesion and myocardial infarction. J Epidemiol Community Health. 2014;68(11):1020–6. 10.1136/jech-2014-204009. 25135074 PMC4600604

[24] Kim ES, Park N, Peterson C. Perceived neighborhood social cohesion and stroke. Soc Sci Med. 2013;97:49–55. 10.1016/j.socscimed.2013.08.001. 24161088

[25] Wethington E, Kessler RC. Perceived support, received support, and adjustment to stressful life events. J Health Soc Behav. 1986;27(1):78–89. 3711634

[26] Dunkel-Schetter C, Bennett TL. Differentiating the cognitive and behavioral aspects of social support. In: Sarason BR, Sarason IG, Pierce GR, editors. Social Support: An Interactional View. New York: John Wiley & Sons, Inc; 1990. pp. 267–96.

[27] Hatzenbuehler ML. Social Factors as Determinants of Mental Health Disparities in LGB Populations: Implications for Public Policy. Social Issues Policy Rev. 2010;4:31–62. 10.1111/j.1751-2409.2010.01017.x.

[28] Todd NR, Nguyễn DM, Blackburn AM, La R. (2024). Associations between state policies and sexual minority mental health disparities. Translational Issues in Psychological Science. Advance online publication. 10.1037/tps0000431

